# The multimodal facilitation effect in human communication

**DOI:** 10.3758/s13423-022-02178-x

**Published:** 2022-09-22

**Authors:** Linda Drijvers, Judith Holler

**Affiliations:** 1 Donders Institute for Brain, Cognition, and Behaviour, Radboud University, Montessorilaan 3, 6525 HR Nijmegen, The Netherlands; 2 Max Planck Institute for Psycholinguistics, Wundtlaan 1, 6525 XD Nijmegen, The Netherlands

**Keywords:** Multimodal communication, Language, Prediction, Audiovisual, Shadowing

## Abstract

During face-to-face communication, recipients need to rapidly integrate a plethora of auditory and visual signals. This integration of signals from many different bodily articulators, all offset in time, with the information in the speech stream may either tax the cognitive system, thus slowing down language processing, or may result in multimodal facilitation. Using the classical shadowing paradigm, participants shadowed speech from face-to-face, naturalistic dyadic conversations in an audiovisual context, an audiovisual context without visual speech (e.g., lips), and an audio-only context. Our results provide evidence of a multimodal facilitation effect in human communication: participants were faster in shadowing words when seeing multimodal messages compared with when hearing only audio. Also, the more visual context was present, the fewer shadowing errors were made, and the earlier in time participants shadowed predicted lexical items. We propose that the multimodal facilitation effect may contribute to the ease of fast face-to-face conversational interaction.

Human face-to-face communication is multimodal. It involves auditory signals, such as speech, as well as visual signals from the body, such as signals conveyed by the hands, face, head, and torso. These auditory and visual signals convey information that unfolds at different moments in time. Such a temporal disalignment may pose a substantial challenge for our language processing system, since for message comprehension a multitude of signals coming from different articulators and modalities have to be combined to form unified, coherent percepts. This cognitive challenge is exacerbated by having to achieve successful message comprehension and response preparation under the tight temporal constraints of conversation (Holler et al., 2018; Levinson, 2016; Stivers et al., 2009). This points toward multimodal language being more effortful to process than unimodal language in continuous discourse (see Chen & Spence, 2018, for corroborating evidence from unimodal and multimodal semantic categorization tasks with visual picture targets), but whether this is indeed so is an open question which the current study aims to address.

There are several domains where evidence has demonstrated that perceiving signals from multiple modalities can lead to facilitation. For example, participants respond faster to multimodal than to unimodal stimuli, such as when presented with a single tone and a light flash compared with just a single tone (e.g., Romei et al., 2007; Senkowski et al., 2006), or a picture (e.g., cow) with an accompanying sound (e.g., “moo”) compared with just a picture (e.g., Molholm et al., 2004; Suied & Viaud-Delmon, 2009; but see Chen & Spence, 2018). Similar results have been observed for speech stimuli: Participants can more accurately recognize degraded or noisy speech when visual speech (e.g., lips) can be seen in addition to hearing sounds (Altieri et al., 2016; Ross et al., 2007). Moreover, although neuroscience studies have demonstrated that visual speech speeds up the neural processing of auditory speech (Arnal et al., 2011; van Wassenhove et al., 2005), there are no studies that have demonstrated a subsequent temporal advantage on behavior (i.e., it is currently unclear whether information from visual speech also causes clear speech to be processed more quickly).

Crucially, the facilitatory effect of multimodal signals is dependent on their integration, which rests on the signals occurring synchronously or within rather small deviations thereof. For low-level auditory and visual signals (beep sounds and light flashes) the temporal binding window extends to around 160 ms (see, e.g., Vidal, 2017; Wallace & Stevenson, 2014, and, with generally more tolerance for asynchronies caused by visual signals leading auditory signals compared with the reverse, Sanders et al., 2019), for speech sounds and visible speech to around 200–300 ms (Munhall et al., 1996; van Wassenhove et al., 2007; Venezia et al., 2016).

However, temporally diverging signal onsets tend to be the norm for human face-to-face communication, and often these divergences are rather large. For example, facial expressions may occur even before any phonation has begun (Kaukomaa et al., 2014), and manual gestures depicting semantic information tend to precede related semantic information in speech often by several hundred milliseconds and up to several seconds (Donnellan et al., 2022; Ferré, 2010; ter Bekke et al., 2020). These temporal distances might make the temporal processing of multimodal utterances harder rather than facilitate it (see Morett et al., 2020, for evidence of this effect for asynchronies between beat gestures and pitch accents). Despite this, asynchronies caused by visual signals leading auditory signals appear to be more easily processed than the reverse (e.g., Cecere et al., 2016; Maier et al., 2011).

Moreover, co-occurring speech and bodily signals such as manual gestures can often convey different information relating to the utterance, meaning that, while they both relate to the same overall message, the information they each encode is far from redundant (e.g., Bavelas et al., 2002; Bavelas et al., 1992; Holler & Beattie, 2003; Kelly et al., 1999). For example, someone might say, “I’m going to the gym,” accompanied with a gesture depicting someone bouncing a ball. While this is not “incongruent” in the sense of conflicting information, this complementarity may nevertheless increase the integration effort that needs to be done, thus increasing processing demands.

Further, processing multimodal signals in human communication is far from trivial since not everything should be integrated. A scratch of the nose is likely not to be communicative and thus should be segregated from the information that is bound together at the message level. Because such signals occur interwoven with meaningful ones, this layer of processing, too, may interfere with a simple multimodal integration heuristic by which processing becomes easier the more signals are present.

However, there is some first evidence indicating that the semantic and pragmatic information conveyed by visual bodily signals might facilitate processing. Studies have shown that participants responded faster to individual word or short-sentence stimuli combined with gestures than to speech-only stimuli (e.g., Holle et al., 2008; Kelly et al., 2010; Nagels et al., 2015), and that questions accompanied by gestures receive faster responses than questions without such gestural components (Holler et al., 2018, ter Bekke et al., 2020). However, the generalizability of these findings is limited due to the experimental findings being based on stimuli involving isolated, carefully acted manual gestures accompanying individual words or scripted sentences. Similarly, findings from corpus studies do not allow for conclusions about causal relations between individual variables.

The present study fills this gap in the literature by testing *the multimodal facilitation hypothesis in human communication* with an experimentally controlled response time task. Crucially, we use a paradigm that combines experimental control with stimuli derived from unscripted face-to-face dialogue. This approach captures and preserves the complex nature of the multimodal signals interlocutors must process in conversation. The stimuli are embedded in a paradigm that uses the classical shadowing task (Marslen-Wilson, 1973). This task is often used to study online language processing and involves participants listening to spoken language which they are asked to repeat as quickly and as accurately as possible. Thus, the task directly involves language comprehension and production, and it directly taps into temporal processing advantages participants might experience in multimodal contexts, as well as into possible effects of visual signals on accuracy.

 More specifically, we asked participants to shadow speech in an audiovisual context (AV), an audiovisual context where the mouth was blurred (AB), and an audio-only context (AO). If the multimodal facilitation hypothesis applies to human communication, we would expect differences between the AO and AV conditions (in both latencies and errors). Further, if this effect is not simply due to the presence of visible speech but also due to visual signals carrying semantic and pragmatic information (e.g., visual bodily signals, such as gestures), we should also observe a difference between the AO and the AB condition.

As an exploratory analysis, we also combined the shadowing paradigm with the Empathy Quotient (EQ) questionnaire (Baron-Cohen & Wheelwright, 2004) to measure whether social sensitivity might affect the propensity for benefitting from multimodal signals during language processing (Eigsti, 2013). Recent studies have demonstrated that participants with a higher EQ are more responsive to visual signals, than participants with a low EQ score (Mandel et al., 2015) also see Hömke et al., 2018, who found a tendency for the effect). Participants with a higher EQ might thus experience a larger advantage from perceiving visual signals.

Finally, the shadowing paradigm offers the additional advantage of also gaining insight into the mechanisms that may be underlying the hypothesized multimodal facilitation effect. It has been postulated that visual cospeech signals may speed up language processing in face-to-face conversation by facilitating prediction (Holler & Levinson, 2019). Prediction is assumed to play a core role in language processing (Huettig, 2015) but has mostly ever been tested with unimodal, spoken/written language stimuli. One exception is a recent EEG study by Zhang et al. (2021), who observed that a neurophysiological marker of prediction in language comprehension (N400) is modulated by the informativeness of co-occurring nonlinguistic signals, such as gestures (i.e., lip movements/meaningful gestures modulated the effect of surprisal on the N400 in response to individual words that they co-occurred with). These results provide evidence that certain visual bodily signals make words more predictable when we encounter them during comprehension. Complementary evidence for visual bodily signals facilitating prediction could be obtained by the shadowing task, since participants in the shadowing task may utter a proportion of the words *before* they hear them—which must be words that they predicted. We thus aim to test whether the hypothesized differences between the experimental conditions also show up in this subset of data which is reflective of predictive cognitive processing. The present study therefore provides a direct test of whether visual bodily signals may facilitate predicting upcoming words during conversational speech and complements extant work (Zhang et al., 2021).

## Method

### Participants

Thirty-seven individuals (mean age = 23.8 years, range: 19–32 years) participated in this experiment. All participants were right-handed, native speakers of Dutch, and reported normal hearing, normal or corrected-to-normal vision, and no language impairments. One participant was excluded from analyses because of familiarity with one of the speakers in the stimuli. The final data set thus consisted of 36 participants. All participants were recruited via the database of the Max Planck Institute for Psycholinguistics database and gave written consent before and after they participated in the experiment. Participants received 10 euros for their participation. The study was approved by the Social Science Ethics Committee of Radboud University.

### Materials

**Fig. 1:**
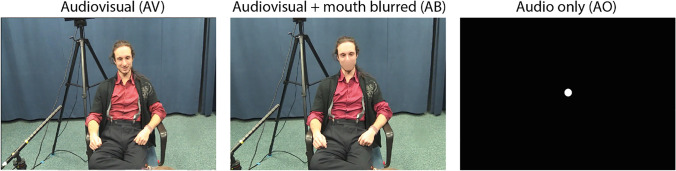
Video stills of conditions

We presented participants with 30 video clips of native Dutch speakers who were engaged in a 1-hour, unscripted, casual conversation with a friend (CoAct corpus, ERC project #773079). Out of these 1-hour conversations, we used ELAN (Wittenburg et al., 2006) to annotate and select 36 video segments in which the speaker would talk about the same topic for about 30 seconds without being interrupted by their conversational partner. In all videos, only one of the two speakers was visible from a frontal perspective. The speaker in the video was always sitting in a chair and was visible from the head to below the knees (see Fig. 1).

We extracted the audio from these video clips, intensity-scaled the speech to 70 dB, and de-noised the speech in Praat (Version 6.0.49; Boersma & Weenink, 2009). All sound files were then recombined with their corresponding video files using Adobe Premiere Pro CC 2018. The videos were presented in three different conditions: audiovisual (AV), audiovisual + mouth blurred (AB), and audio only (AO). The AB condition was created by manually blurring all visual speech of a speaker using Adobe Premiere Pro CC 2018. This was done on a frame-by-frame basis to ensure that the blur would dynamically fit the speaker’s mouth, even when the speaker would be moving in the video. In the AO condition, the participants only heard the speech of the video clips while they saw a white dot on the screen.

#### Empathy Quotient questionnaire

Participants were asked to complete a Dutch version of the Empathy Quotient test to measure empathy (Cronbach’s alpha = 0.9; Baron-Cohen & Wheelwright, 2004). Participants filled out the 60-item questionnaire by rating how much they agreed or disagreed with each statement on a 4-point Likert scale (ranging from *strongly agree* to *strongly disagree*). Depending on their answer, a participant could score 2, 1, or 0 on an item (following a scoring key from Baron-Cohen & Wheelwright, 2004). Forty items in the questionnaire measured empathy, and 20 were fillers. A participant could thus maximally score 80 points.

### Procedure

All participants first received verbal and written instructions about the entire testing procedure. We then acquired informed consent for the experiment and asked participants to complete the Empathy Quotient questionnaire, which took approximately 5 minutes. (Participants were also asked to complete a number of additional questionnaires not of relevance regarding the present analyses; this included the digit span task, the letter fluency task, the trail-making test, and the animal fluency task).

After a short break, participants commenced with the main experiment. Participants were tested in a soundproof booth and seated in front of a computer with headphones on. In the booth, we set up a webcam to monitor participants’ visual attention, as well as a microphone to record participants’ speech. We instructed participants to closely shadow the speech in the audio/video stimuli as soon as each audio/video clip started, and to press the space bar at the end of the clip to move on to the next trial.

All videos were presented using Presentation (Neurobehavioral Systems) and displayed on a 24-inch monitor with 1,920 × 1,080-pixel resolution. All stimuli were presented in a blocked design, and the order of blocks and the items within them were randomized over participants. Each block contained 10 stimulus clips. Before the experimental trials of a new block started, participants completed two practice trials in that specific condition to get familiar with the stimuli of the upcoming block. Participants only saw each stimulus once, as none were repeated over conditions. As soon as the clips started, the speech recording was also initiated. Any delays between the start of the video and the start of the speech recording were corrected for in our analysis, and never consisted of more than 15 ms. After every block, participants could take a self-paced break. The main experiment lasted for about 30 minutes.

After the main experiment, the participants again filled out a short questionnaire about their demographics and were debriefed about the experiment.

### Shadowing analyses

#### Preprocessing

In order to analyze the recorded speech from the participants, we annotated all words and their word boundaries per stimulus clip*.* This was necessary to accurately calculate shadowing latencies and shadowing errors in our main analyses. Specifically, we used an automatic speech recognizer (van den Bosch et al., 2007) to annotate all words, and then manually corrected the words and their onset and offset boundaries in Praat. A similar approach was used for the 1,080 speech output files of the participants. Out of these 1,080 files, 15 files were excluded from the analysis because the participant accidentally pressed the space bar to move on to the next trial too early. This resulted in 1,065 files that were used in our analyses, containing 112,412 words. All data and analyses are available on OSF (https://osf.io/nu8mj/).

#### Error analysis and shadowing latencies analysis

We analyzed the annotated and corrected speech output in MATLAB (Version 2016b). Here, we first loaded a participant’s output file and the corresponding stimulus file. We then listed all spoken words in the original stimulus file and the participant’s output file, as well as their onsets. In short, errors were defined as all words that the participant did not utter, but which did occur in the original stimulus file, as well as all words that the participant uttered, but which did not occur in the original stimulus file. This included hesitations, repetitions, mispronunciations, stutters, and slips of the tongue. When a word was correct, we calculated its shadowing latency by subtracting the timing of the onset of the word in the original stimulus file from the onset of the shadowed word in the participant’s output file.

### Statistical analyses

We tested whether the percentage of shadowing errors and shadowing latencies were predicted by the condition (AO/AB/AV) in which the stimulus was presented. For the exploratory analysis, in a second step, we tested whether EQ scores affected accuracy and shadowing latency per condition. As a control variable, we added the speech rate of the stimuli as a fixed effect to all models. Previous work demonstrated that speech rate affects how well recipients can shadow speech (van Paridon et al., 2019), and since all of our stimuli were derived from natural conversations, the speech rate in our stimuli was not experimentally controlled for.

We fitted Bayesian linear mixed models using the *brms* package (Version 2.14.0; Bürkner, 2017) in R (Version 4.0.1; R Core Team, 2020). For the accuracy data, we used a Gaussian distribution with an identity link function and included a full random effects structure (both intercepts and slopes for participants and items). We set weakly informative priors for all coefficients (normal distribution centered on zero, standard deviation of two, assuming that most effects are small), and for all group-level random effects, we used the default priors set by the *brms* package.

For the reaction times data, we used a student’s *T* distribution and identity link function, and again included a full random effects structure (both intercepts and slopes for participants and items). We used the default priors set by the brms package for all coefficients and group-level random effects. Both models were fit using Markov Chain Monte Carlo and a No-U-Turn-Sampler. All parameters had a Gelman–Rubin statistic Rhat equal to 1.0. Next to mean estimates, standard errors and 95% credible intervals for all fixed effects model parameters, we report Bayes factors quantifying how much more likely the data are under the alternative hypothesis than under the null hypothesis (BF10, following interpretation of Jeffreys, 1961). We used the hypothesis function of the brms package to calculate an evidence ratio for each hypothesis. This evidence ratio is the ratio of the posterior probability of a > b and the posterior probability of a < b, following Bürkner (2017).

## Results

### The more visual signals were present, the fewer errors participants made during speech shadowing

Out of all shadowed words, 39.43% (*SD* = 13.97) were incorrect in the AO condition, 38.07% (*SD* = 13.58) were incorrect in the AB condition, and 35.81% (*SD* = 12.88) were incorrect in the AV condition. Participants made more omission errors than addition errors, and this pattern was consistent over conditions (omissions: AO: 31.75%, AB: 30.78%, AV: 28.79% of all errors, additions: AO: 7.68%, AB: 7.29%, AV: 7.02%).

Participants made fewer errors in the AV than the AO condition, coefficient posterior *M* = −2.88, *SE* = .61, CI [−3.88, −1.86], BF_10_ = Inf, corresponding to very strong evidence, posterior probability 100%, fewer errors in the AB than the AO condition, *M* = −1.29, *SE =* −.55*,* CI [−2.18, −.38], BF_10_ = 101.56 (very strong evidence), posterior probability 99%, and fewer errors in the AV than the AB condition, *M* = −1.59, *SE* = .62, CI [−2.61, −.55], BF_10_ = 149.94 (very strong evidence), posterior probability 99%.

**Fig. 2:**
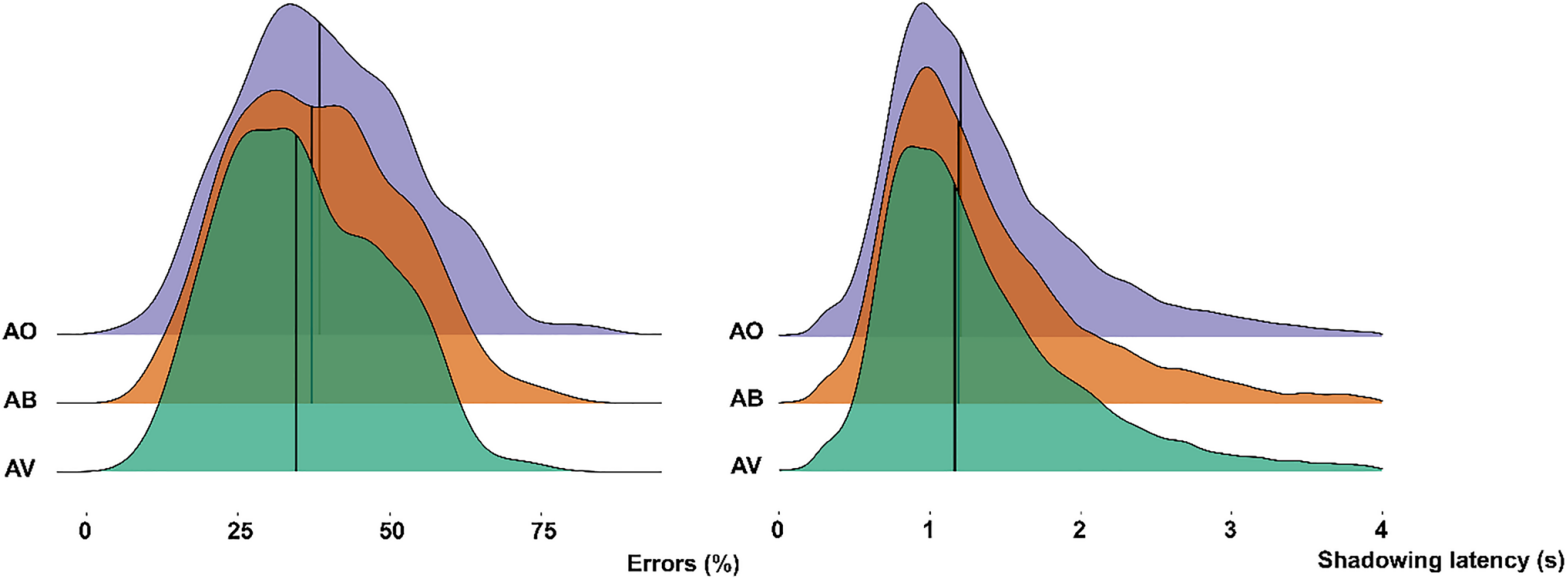
Stacked density plots for each condition of % of errors per condition (left) and of shadowing latencies per condition (right). The line represents the median. Participants make less errors in the AV condition versus the AB and AO condition (AV < AB < AO) and are quicker in shadowing in the AV condition than in the AB and AO condition (AV < AB = AO). (Color figure online)

**Fig. 3:**
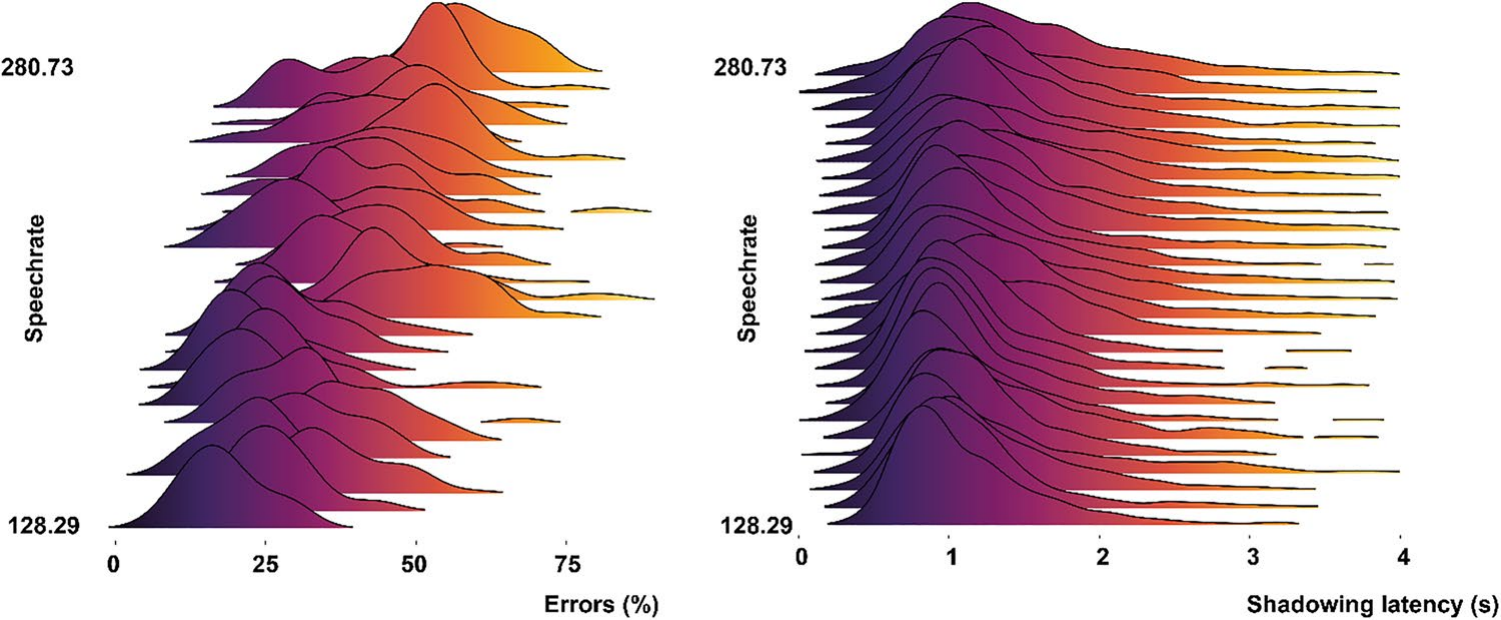
Joy plots of error distribution per speech rate (left), and shadowing latency distribution per speech rate (right). Low speech rates are associated with less errors and faster shadowing latencies than high speech rates. (Color figure online)

These results demonstrate that the more visual signals were present, the less participants made errors in speech shadowing (see Fig. 2). Participants were more likely to make an error when speech rate increased (*M* = 5.27, CI [2.76, 7.44]; see Fig. 3).

### The more visual signals were present, the more strongly EQ affected the amount of errors during speech shadowing

**Fig. 4:**
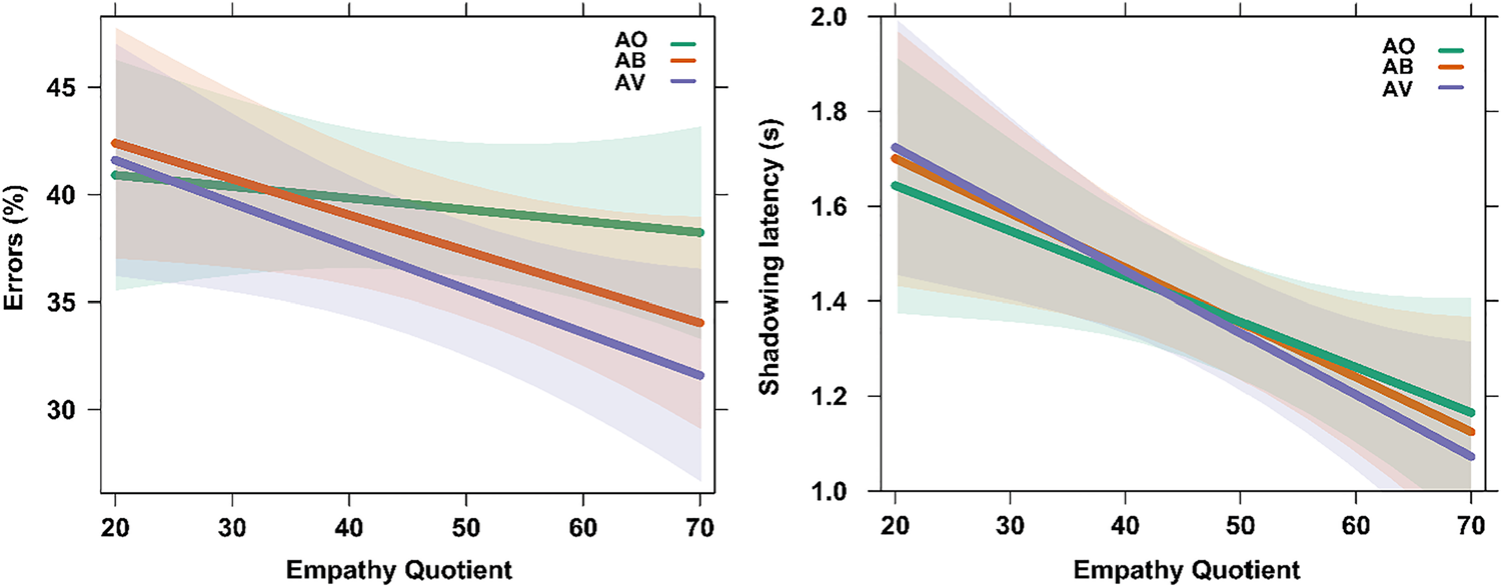
EQ test scores by error percentages and shadowing latency, per condition. The effect of EQ test score on error % and shadowing latencies was strongest in the AV condition (AO < AB < AV). Shaded areas represent the Bayesian credible interval. (Color figure online)

Participants’ EQ score affected the amount of errors made: the effect of EQ was stronger in the AV compared with the AO condition, *M* = −1.61, *SE =* .55*,* CI [−2.51, −.71], BF_10_ = 443.44 (very strong evidence), posterior probability 100%, stronger in the AB than the AO condition, *M* = −1.22, *SE =* .51, CI [−2.05, −0.39], BF_10_ = 101.56 (very strong evidence), posterior probability 99%, and stronger in the AV than the AB condition, *M* = −.38, *SE* = .57, CI [−1.32, .54], BF_10_ = 3.07 (substantial evidence), posterior probability 75%. The higher the EQ score, the fewer errors were made, especially when all visual signals were present in the videos (see Fig. 4).

### When all visual signals were present, participants shadowed speech faster

On average, participants shadowed words with a latency of 1,364 ms in the AO condition (*SD* = 766 ms), 1,383 ms in the AB condition (*SD* = 819 ms), and 1,331 ms in the AV condition (*SD* = 768 ms; see Fig. 1).

We observed faster shadowing latencies in the AV compared with the AO condition, *M* = −.02, *SE =* .02, CI [−.05, .01], BF_10_ = 4.52, (substantial evidence), posterior probability 82%, but not when comparing AB to AO, *M* = −.01, *SE* = .02, CI [−.05, .02], BF_10_ = 2.81 (anecdotal evidence), posterior probability 74%, or AV to AB (*M* < 0.01, *SE* = .03, CI [−.05, .04], BF_10_ = 1.24, posterior probability 55%. Participants were more likely to have a slower shadowing latency when speech rate increased (*M* = .1, *SE* = .01, CI [.07, .13]; see Fig. 3).

### The more visual signals were present, the more strongly EQ affected shadowing latency

Finally, we observed an effect of EQ score on shadowing latencies (see Fig. 4). EQ score affected shadowing latencies more strongly in the AV than the AO condition, *M* = −.04, *SE* = .02, CI [−.07, −.01], BF_10_ = 134.59 (very strong evidence), posterior probability 99%, more strongly in the AB than the AO condition, *M* = −.02, *SE* = .02, CI [−.05, .02], BF_10_ = 3.5 (substantial evidence), posterior probability 78%, and more strongly in the AV than the AB condition, *M* = −.02, *SE* = .02, CI [−.06, .02], BF_10_ = 5.08 (substantial evidence), posterior probability 84%. In general, a higher EQ score was associated with faster shadowing latencies, especially when more visual signals were present.

### Does the presence of visual signals affect the shadowing of words before they are heard?

Finally, we investigated whether we could specify the cognitive mechanisms that may underlie the multimodal facilitation effect, by zooming in on words that participants uttered *before* they heard them. We therefore retained only those lexical items uttered with a shadowing latency under 0 ms—that is, words that were uttered before participants encountered them in our stimuli. These lexical items must have been anticipated (i.e., predicted) on the basis of the preceding context.

We found that 1,211 words were uttered before participants encountered them, corresponding to ~1.2% of all data. This number was equal over conditions (AO: 405, AB: 400, AV: 406). Participants anticipated words most strongly in the AV condition (*M* = −792 ms, *SD* = 44 ms), followed by the AB (*M* = −781 ms, *SD* = 45 ms) and the AO condition (*M* = −769 ms, *SD* = 45 ms).

**Fig. 5:**
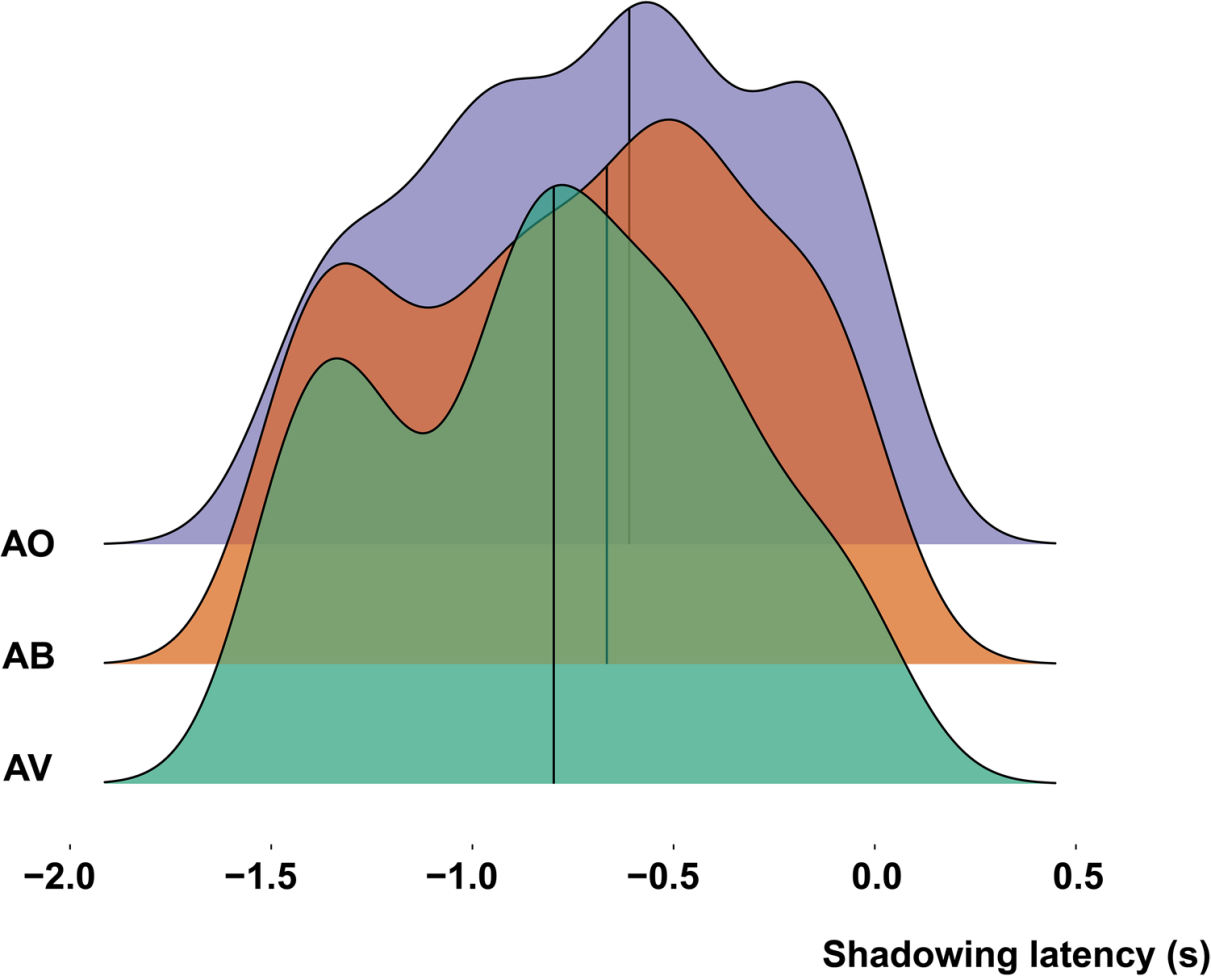
Stacked density plots for each condition of shadowing latencies of content words that were uttered before participants heard them. The line represents the median. Participants utter predicted words earlier in the AV condition

As an exploratory post-hoc analysis, we reasoned that this effect was probably even more prominent for content words since content words might be easier to predict than function words. Here we observed a similar pattern, with strongest anticipation in the AV condition (*M* = −811 ms, *SD* = 42 ms), followed by the AB (*M* = −750 ms, *SD* = 44 ms), and the AO condition (*M* = −691 ms, *SD* = 43 ms; see Fig. 5).

Participants anticipated words earlier in time in the AV than the AO condition, *M* = −.1, *SE =* .05, CI [−.18, .01], BF_10_ = 26.12 (strong evidence), posterior probability 96%, and anticipated words earlier in time in the AV than the AB condition, *M* = −.04, *SE =* .06, CI [−.14, .06], BF_10_ = 3.08, posterior probability 75%. Finally, we found substantial evidence that participants anticipated words more strongly in the AB than the AO condition, *M* = −.05, CI [−.14, .03], BF_10_ = 5.64, posterior probability 85%.

In summary, these results demonstrate that participants anticipated words before they heard them in all conditions, but that this effect was stronger the more visual signals are present.

## Discussion

The inherently multimodal nature of human face-to-face communication confronts us with the question of how interlocutors manage to process and rapidly integrate the plethora of visual and vocal signals during conversation, despite their differing onsets and despite having to filter the signal stream based on relevance. While unification appears highly challenging in the light of how multimodal utterances are composed and embedded, we here found evidence for a multimodal facilitation effect in naturalistic human communication: participants were faster and more correct at shadowing speech when they perceived utterances in their multimodal entirety rather than the unimodal, verbal equivalents. Critically, we observed effects attributable to both, visible speech as well as other visual signals.

These results extend the well-established multisensory facilitation effects found in other domains, such as with simpler, low-level stimuli (e.g., Molholm et al., 2004; Romei et al., 2007; Senkowski et al., 2006; Suied & Viaud-Delmon, 2009), to the domain of communication. Evidence of multimodal facilitation has been obtained with communication-related stimuli before, such as lips and sounds (e.g., Altieri et al., 2016), but previous work has solely reported a gain in accuracy when observing lip movements in clear speech. Here, we present first evidence that such a multimodal facilitation effect also gives rise to a *temporal* advantage in clear speech. Crucially, the findings demonstrate an overall *multimodal facilitation effect* in the context of the complex, multilayered, multimodal utterances that interlocutors produce and perceive in face-to-face communication. As such, they complement recent work by Zhang et al. (2021) who showed that the presence of lip movements and meaningful manual gestures modulated the surprisal values of words they accompanied, thus facilitating processing. Moreover, the fact that we observe an overall multimodal facilitation effect suggests that multiple visual signals might contribute to this facilitation and underlines the importance of studying language in its rich, naturalistic context, a feat that is often ignored in multimodality research that focuses on the contribution of a single visual signal (e.g., hand gestures).

An important question is what causes this multimodal facilitation effect in human communication. Temporal convergence has been shown to be a key factor in multisensory integration for many types of visual-auditory signals (Munhall et al., 1996; Venezia et al., 2016; van Wassenhove et al., 2007). Due to the timing being far from synchronous for the visual and vocal signals constituting complex utterances in face-to-face interaction, temporal convergence is likely to be playing a more minor role in explaining the multimodal facilitation effect that we found, if any at all. Holler and Levinson (2019) have proposed that both low-level statistical associations as well as higher-level semantic analysis is likely to be influencing the binding of multimodal signals into unified utterances. Crucially, they argue that prediction, in combination with bilateral processes between those levels, is one of the fundamental mechanisms on which signal integration in face-to-face conversation is based.

Here, we obtained experimental evidence that visual bodily signals play a role in language prediction. Participants uttered a proportion of correct words before they heard them, and this was more pronounced for multimodal than unimodal utterances. Specifically, the more visual information was present, the more participants engaged in predictive language processing. One possibility is that this is due to the visual signals aiding prediction by themselves, such as through seeing a facial signal or manual gesture foreshadowing semantic or pragmatic information derivable from the unfolding verbal utterance only further “downstream,” thus speeding up the shadowing of words in the present task. This would be in line with Holler and Levinson’s (2019) hypothesis. Alternatively, visual signals may not be predictive in themselves but may be facilitating linguistic predictions by speeding up linguistic processing in general. For example, the presence of biological motion during perception may heighten attention, or the visual presence of a speaker may make perceivers feel more engaged and socially motivated. And, of course, such explanations are not mutually exclusive with the possibility that visual signals themselves are predictive of upcoming semantic or pragmatic content since these processes may work in unison. Future research is needed to tease apart the individual contributions of these possible mechanisms, as well as the individual contributions of the different visual signals involved in this process (e.g., gestures, body movements, facial cues). Especially manual gestures conveying semantic or pragmatic information may enhance predictive effects further (ter Bekke et al., 2020), but this possibility requires experimental testing (the current corpus-based data set did not yield large enough numbers in this respect). Moreover, the (temporal) relationship of visual and auditory information differs per signal: In the AV condition, lip movements are temporally close to speech, and directly relate to the sensory properties of the speech signal. This is not necessarily the case for other visual bodily signals in the AV/AB condition, depending on their type: Manual gestures (e.g., iconic or pragmatic gestures) and facial expressions often complement speech semantically and/or pragmatically and often precede corresponding speech. They thus stand in a different temporal and functional relationship to the speech signal. The extent to which multimodal signal processing involves integration and prediction is therefore likely to vary by signal type

Lastly, we investigated whether social sensitivity, as measured by the Empathy Quotient, might affect the propensity for benefitting from multimodal signals during language processing. Indeed, we observed that a higher EQ score was associated with fewer errors and shorter shadowing latencies, especially when more visual signals were present. We propose that participants with a higher EQ score may be more responsive to visual information than participants with a lower EQ score, allowing them to benefit more from these signals during language processing. Future studies are needed to follow up on this proposal with a larger sample size, and to more systematically study the effects of EQ on multimodal language comprehension. Future research may also shed light on the potential link between the role of empathy and multimodal language processing in autism spectrum disorder.

The present study is limited in that the shadowing paradigm does not allow us to disentangle multimodal facilitation effects relating to comprehension versus production (for entrainment effects in production, see Fridricksson et al., 2012), thus requiring future experimental studies teasing these effects apart. The paradigm also does not fully capture communication in situ. While the stimuli used in this study are longer extracts taken from natural, unscripted face-to-face conversation, participants were able to view but not interact with the speakers. Furthermore, the AB condition might have resulted in a speed–accuracy trade-off: While accuracy was higher in the AB than the AO condition, latencies were longer, possibly due to a higher processing load that was induced by the blurred face (after all, this condition corresponds to the least natural form of communication compared with face-to-face [AV] or telephone [AO]). Thus, one crucial next step for future studies is to embed the current paradigm in social interaction to establish the generalizability of the multimodal facilitation effect. Moreover, the shadowing process in itself explicitly asks participants to repeat what they hear, which does not occur often in natural conversations. However, if anything, it is likely that the current study is underestimating the strength of this effect. In an immersed environment with life-sized multimodal stimuli and personal copresence, plus the actual involvement in the conversation, we would expect the effects we found to be even more pronounced.

To conclude, we have here demonstrated a multimodal facilitation effect in human communication, for the first time taking as a basis the complex, rich multimodal utterances consisting of many layers of vocal and visual signals, all temporally distributed in time. This finding significantly advances our understanding of how interlocutors in face-to-face conversation deal with this complexity in the rapid environment of conversation. Moreover, the shadowing paradigm employed here provides a first glimpse of the more detailed mechanisms that may be underpinning the multimodal facilitation effect, of which prediction, or at least the facilitation of predictive processes, seems to be one important component.
